# Nanometric axial resolution of fibronectin assembly units achieved with an efficient reconstruction approach for multi-angle-TIRF microscopy

**DOI:** 10.1038/s41598-018-36119-3

**Published:** 2019-02-13

**Authors:** Emmanuel Soubies, Agata Radwanska, Dominique Grall, Laure Blanc-Féraud, Ellen Van Obberghen-Schilling, Sébastien Schaub

**Affiliations:** 1 Université Côte d’Azur, CNRS, Inria, I3S France; 2 Université Côte d’Azur, CNRS, Inserm, iBV France; 3 Centre Antoine Lacassagne, Nice, France; 4 Present Address: Biomedical Imaging Group, EPFL, Lausanne, Switzerland

## Abstract

High resolution imaging of molecules at the cell-substrate interface is required for understanding key biological processes. Here we propose a complete pipeline for multi-angle total internal reflection fluorescence microscopy (MA-TIRF) going from instrument design and calibration procedures to numerical reconstruction. Our custom setup is endowed with a homogeneous field illumination and precise excitation beam angle. Given a set of MA-TIRF acquisitions, we deploy an efficient joint deconvolution/reconstruction algorithm based on a variational formulation of the inverse problem. This algorithm offers the possibility of using various regularizations and can run on graphics processing unit (GPU) for rapid reconstruction. Moreover, it can be easily used with other MA-TIRF devices and we provide it as an open-source software. This ensemble has enabled us to visualize and measure with unprecedented nanometric resolution, the depth of molecular components of the fibronectin assembly machinery at the basal surface of endothelial cells.

## Introduction

Fluorescence microscopy is widely used in biology to observe selected molecules and subcellular structures of interest. However, the resolution of conventional techniques (e.g., widefield, confocal) is limited by the diffraction phenomenon. Since the early 90’s, this limitation has been overcome by super-resolution techniques, achieving unprecedented nanoscale resolution. Single molecule localization microscopy (SMLM) techniques^1,2^ rely on photoactivatable fluorescent probes together with PSF engineering and dedicated localization algorithms. Other methods, such as structured-illumination microscopy (SIM)^3,4^, use standard staining but specific excitation processes which move high-frequency content into the observable region of the microscope. A numerical reconstruction from a set of SIM acquisitions with varying illuminations can double the resolution of conventional systems.

Total internal reflection fluorescence (TIRF) microscopy offers a unique optical sectioning of areas adjacent to the glass coverslip. This technique, introduced by Axelrod in the 80’s^5^, relies on an evanescent excitation produced in the total internal reflection regime. The fast decay of the evanescent field in the axial direction limits the observed region to a thin layer of a few hundred nanometers. This property makes TIRF microscopy ideally suited to the observation of biological activities near the cell membrane^6^. Moreover, an important advantage is that TIRF microscopy does not require any specific dyes, PSF engineering, or complex excitation processes, but it only requires a tilted illumination beam. This leads to high-quality (low out-of-focus signal and high signal-to-noise ratio) and fast live imaging of cell/substrate interactions.

**Figure 1:**
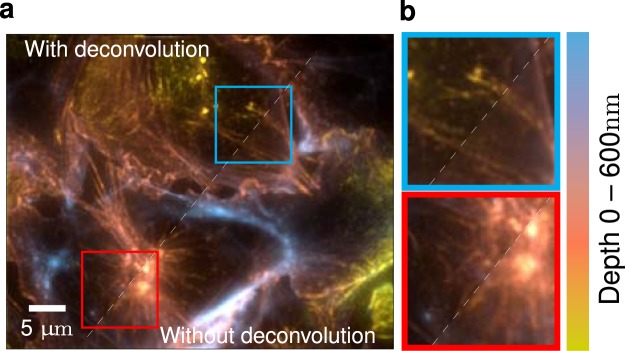
MA-TIRF reconstruction of actin filaments. The colormap encodes the axial position of the reconstructed structures from 0 to 600 nm. (**a**) Filamentous actin in cultured endothelial cells is stained with Alexa Fluor 488-conjugated phalloidin. Comparison of reconstructions with and without considering the convolution operator in the model. (**b**) Differences are further exemplified in the zoomed regions.

Although a single TIRF image does not provide a quantitative axial information, multi-angle TIRF (MA-TIRF) acquisitions can be used to estimate the depth of biological structures using dedicated reconstruction algorithms (see Fig. 1). Most of the existing reconstruction methods are based on a shape prior (model) to extract the axial depth of vesicles^7–10^, membranes^11–18^, or microtubules^19,20^. Estimation of model parameters is generally performed via curve fitting^7,8,11–14,20^, or by exploiting the ratio between a TIRF and a widefield acquisition^15–17^ or two TIRF images^18^. Other works have considered Bayesian estimation^9,19^ or marked point processes^10^. This vast literature contrasts with the limited number of studies dealing with full three-dimensional reconstruction. In particular, variational methods dedicated to solving the inverse problem using adequate constraints and sparse regularizations. To our knowledge, these are limited to the work of Boulanger *et al*.^21^ as well as the recent study by Zheng *et al*.^22^. In this paper, we propose a new reconstruction approach for MA-TIRF microscopy that performs jointly depth estimation and lateral deconvolution.

This novel high resolution, multi-wavelength MA-TIRF imaging approach offering quantitative axial information (up to 400 nm) is well suited for analyzing integrin-dependent events that take place at the cell-substrate interface. Integrins are transmembrane receptors for the extracellular matrix that play essential roles in cell adhesion, survival, and migration^23^. Following ligand binding to their extracellular domain, integrins trigger the formation at their cytoplasmic tails of large adhesion complexes, termed focal adhesions, comprised of key adaptor proteins and signaling molecules^24^. We have previously identified the cytoplasmic adaptor Integrin-linked kinase (ILK) as an obligate partner of integrin *α*5*β*1 for fibrillar assembly of fibronectin beneath endothelial cells^25^. Here we used MA-TIRF imaging of integrin *α*5*β*1, ILK, and fibronectin to obtain further topographical and temporal insights into this process. The extended range of axial measurements achieved with our MA-TIRF system was critical for this study, as extracellular deposition of fibronectin accumulates with time. Beyond fibronectin fibrillogenesis, our MA-TIRF setup should be valuable for addressing emerging questions relating to integrin-mediated regulation of cell adhesion, actin and microtubule cytoskeletons, membrane transport and signal transduction.

## Results

### A Fast and Flexible MA-TIRF Pipeline

One of the main novelties of the proposed approach concerns the joint reconstruction and deconvolution which improves the lateral quality (deconvolution) while reconstructing super-resolved information in the axial direction. The achieved improvement can be appreciated in the height map shown in Fig. 1, which depicts actin fibers at the basal surface of cultured endothelial cells. The actin filaments are considerably more contrasted when the joint estimation algorithm is deployed.

**Figure 2:**
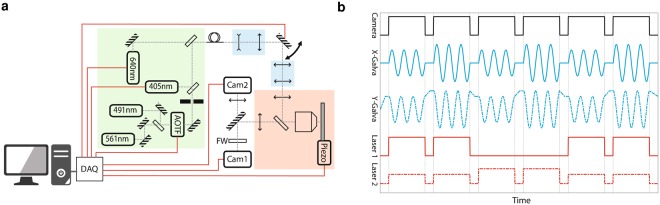
Multi-Angle TIRF system. (**a**) The opto-electronic scheme. Fibered laser bench (green) is coupled to the microscope (red) through galvanometric mirrors and two beam expanders (blue) to achieve homogeneous illumination and accurate illumination angles. All fast electronic devices are synchronized by the DAQ optimizing the acquisition rate. (**b**) The camera transfer time (1.5 ms) is exploited to move the galvanometric mirrors to their subsequent locations.

Regarding the proposed setup (Fig. 2), one key element is the scanning of the back focal plane. With a direct camera control, we tune and insure the angular precision of the lasers, which is required to control the illumination depth. The acquisition speed is optimized by electronical synchronization using a dedicated data acquisition device (DAQ) card (Fig. 2a). We set the camera in “frame transfer mode” to strongly reduce the time of transfer to the computer. Moreover, we took advantage of the lag time between the exposures to pre-position the laser angle with the galvanometric mirrors (two mirrors are used to provide conical illumination)^21^. For multi-channel acquisitions, we changed the filter between the angle series (a multi-band filter is also available for fast acquisition). This setup allows the recording of MA-TIRF data for tri-dimensional reconstruction (≤10 angles) in less than one second per channel.

### Three-Dimensional Reconstruction

The image formation process through a TIRF microscope follows the model

1 $${[{\bf{g}}(\alpha )]}_{i}={\int }_{{\bf{r}}\in {{\rm{\Omega }}}_{i}}{\int }_{{{\bf{r}}}^{\text{'}}\in {\rm{\Omega }}}{\int }_{z=0}^{+\infty }h({\bf{r}}-{\bf{r}}{\boldsymbol{^{\prime} }},\,z-{z}_{{\rm{fp}}})\,I\,(z,\,\alpha )\,f\,({\bf{r}}{\boldsymbol{^{\prime} }},\,z)\,{\rm{d}}z\,{\rm{d}}{\bf{r}}{\boldsymbol{^{\prime} }}\,{\rm{d}}{\bf{r}}+{b}_{i}\mathrm{.}$$

Here, *α* is the incident angle of the illumination beam, **r** = (*x*, *y*) ∈ Ω corresponds to the lateral variable in the image domain ${\rm{\Omega }}\subset {{\mathbb{R}}}^{2}$, and ${{\rm{\Omega }}}_{i}\subset {\rm{\Omega }}$ is the region of the image domain that corresponds to the ith pixel. The excitation field *I*(*z*, *α*) varies with both *α* and *z* according to an exponential law $I(z,\,\alpha )={I}_{0}(\alpha ){e}^{-zp(\alpha )}$ where *I*_0_(*α*) and *p*(*α*) are the intensity at the interface (*z* = 0) and the inverse of the penetration depth of the evanescent wave, respectively^5,26,27^. Finally, *h* is the PSF of the device (diffraction), *z*_fp_ > 0 the position of the focal plane, and *b*_*i*_ ≥ 0 models the background signal level for the ith pixel. More details are provided in *Methods* and *Supplementary Information: TIRF Theory*.

Despite its high optical sectioning capability at the vicinity of the cell membrane, TIRF microscopy does not provide quantitative axial information on the three-dimensional density of fluorophores $f:{\rm{\Omega }}\times {{\mathbb{R}}}_{\geqslant 0}\to {{\mathbb{R}}}_{\geqslant 0}$. However, such a quantitative estimation is made possible from a set of TIRF acquisitions for different incident angles *α* (MA-TIRF) by developing dedicated reconstruction algorithms. We solved this inverse problem (*i.e*., estimating *f* in (1) from multi-angle acquisitions ${\bf{g}}={\{{\bf{g}}({\alpha }_{m})\}}_{m\in \mathrm{[1}\ldots M]}$) through a variational approach. This allows the inclusion of relevant constraints to the solution such as nonnegativity and spatial regularity which are essential to deal with the ill-posedness of the problem. Formally, we aimed at solving the optimization problem

2 $$\hat{{\bf{f}}}={\rm{\arg }}\mathop{{\rm{\min }}}\limits_{{\bf{f}}\in {{\mathbb{R}}}_{\geqslant 0}^{{N}_{{\rm{xy}}}\times {N}_{{\rm{z}}}}}\frac{1}{2}{\Vert {\bf{T}}{\bf{H}}{\bf{f}}-{\bf{g}}\Vert }_{2}^{2}+\mu R({\bf{L}}{\bf{f}}),$$

where ${\bf{f}}\in {{\mathbb{R}}}_{\geqslant 0}^{{N}_{{\rm{xy}}}\times {N}_{{\rm{z}}}}$ and ${\bf{T}}{\bf{H}}:{{\mathbb{R}}}_{\geqslant 0}^{{N}_{{\rm{xy}}}\times {N}_{{\rm{z}}}}\to {{\mathbb{R}}}_{\geqslant 0}^{{N}_{{\rm{xy}}}\times M}$ are discrete versions of the three-dimensional density of fluorophore *f* and the forward model (1), respectively. The objective function in (2) is the sum of a data fidelity term (measuring the discrepancy between the model **THf** and the data **g**) and a regularization functional $\mu R({\bf{L}}\cdot )$. We deployed an efficient and flexible algorithm to solve problem (2) which relies on the alternative direction method of multipliers (ADMM)^28^. It can be used with several regularizers *R* such as the order-one Schatten-norm of the Hessian operator^29^, or the popular total variation (TV)^30^. As an illustration of its speed, the reconstruction of a (512 × 512 × 15) volume with a TV prior takes 30 s. Moreover, having access to a graphic processing unit (GPU), the algorithm can be easily switched to a mode which benefits from this parallel device to improve speed by a factor of ten (*i.e*., 3 s). A complete description of the algorithm, detailing the splitting strategy as well as the explicit form of each sub-problem, is provided in *Methods* and *Supplementary Information*.

### Calibration and model validation

Relevant reconstructions are made possible only with a proper calibration of the incident angles used during the acquisition. To that end, we deployed an approach that we previously proposed in a preliminary communication^31^. It involves estimating the relation linking the tension applied to the galvanometric mirror and the incident angle, from the recording of the objective back focal plane. The latter exhibits a ring whose radius is directly related to the incident angle^16,21,31^.

**Figure 3:**
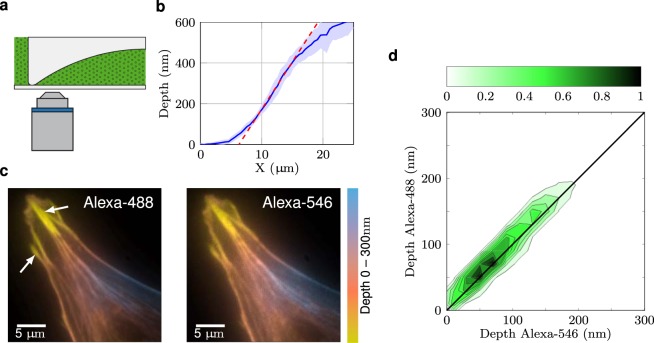
Model validation. (**a**) A fluorescent liquid (dotted green area) between the coverslip and a divergent spherical lens was used as a known phantom sample to validate the model. (**b**) The estimated lens profile from MA-TIRF acquisitions follows the expected slope (dashed red line) of the lens within the observed region. (**c**) Color-coded depth representations of two independent acquisitions and reconstructions of the same biological sample (endothelial cells co-stained with Alexa-561- and Alexa-488-coupled phalloidin) reveal an accurate co-localization. White arrows mark adhesion sites. (**d**) The robust co-localization is further demonstrated in the 2D histogram reporting the relative depth between the two fluorophores, which exhibits an almost perfect diagonal pattern.

Then, in order to estimate the accuracy of the setup, we imaged a fluorescent liquid (fluorescein isothiocyanate) between the coverslip and a divergent spherical lens with radius of curvature R = 288 mm and diameter $\varnothing$ = 25,1 mm (Fig. 3a). The reconstruction algorithm, based on curve fitting and top-hat shape prior^31^, extracted the thickness of the fluorescent layer (Fig. 3b). This showed a linear behavior in the range of 100 nm to 400 nm in depth, which is consistent with the theoretical slope (dashed red line). The deviation of the experimental results from the theoretical slope under 100 nm can be explained by the bevel on the lens border. Above 400 nm, the reconstructed profile also deviated from the expected slope, reflecting the difficulty in recovering deeper structures. It is noteworthy that such a reconstruction of a known phantom up to 400 nm has not been reported to date. Previous studies have generally presented estimation results for the first 200 nm^14,21^.

To compare localization of different dyes in a single sample, it is also crucial for the setup to be achromatic. To test this, cells were stained with a mixture of phalloidin coupled to Alexa-561 or Alexa-488. Data were acquired and reconstructed independently. The results are strikingly similar in the two channels, as shown by the color-coded depth representations (Fig. 3c). This accurate co-localization is corroborated by the 2D-histogram (Fig. 3d), indicating the relative depth between phalloidin in the two channels, which shows an almost perfect diagonal pattern. Finally, as expected, actin fibers were in close contact with the coverslip at focal adhesions near the termini of stress fibers (white arrows), whereas their nanometric height increased towards the center of the cell.

### Axial resolution and mean depth determination of adhesion-associated proteins in endothelial cells on a fibronectin-coated substratum

**Figure 4:**
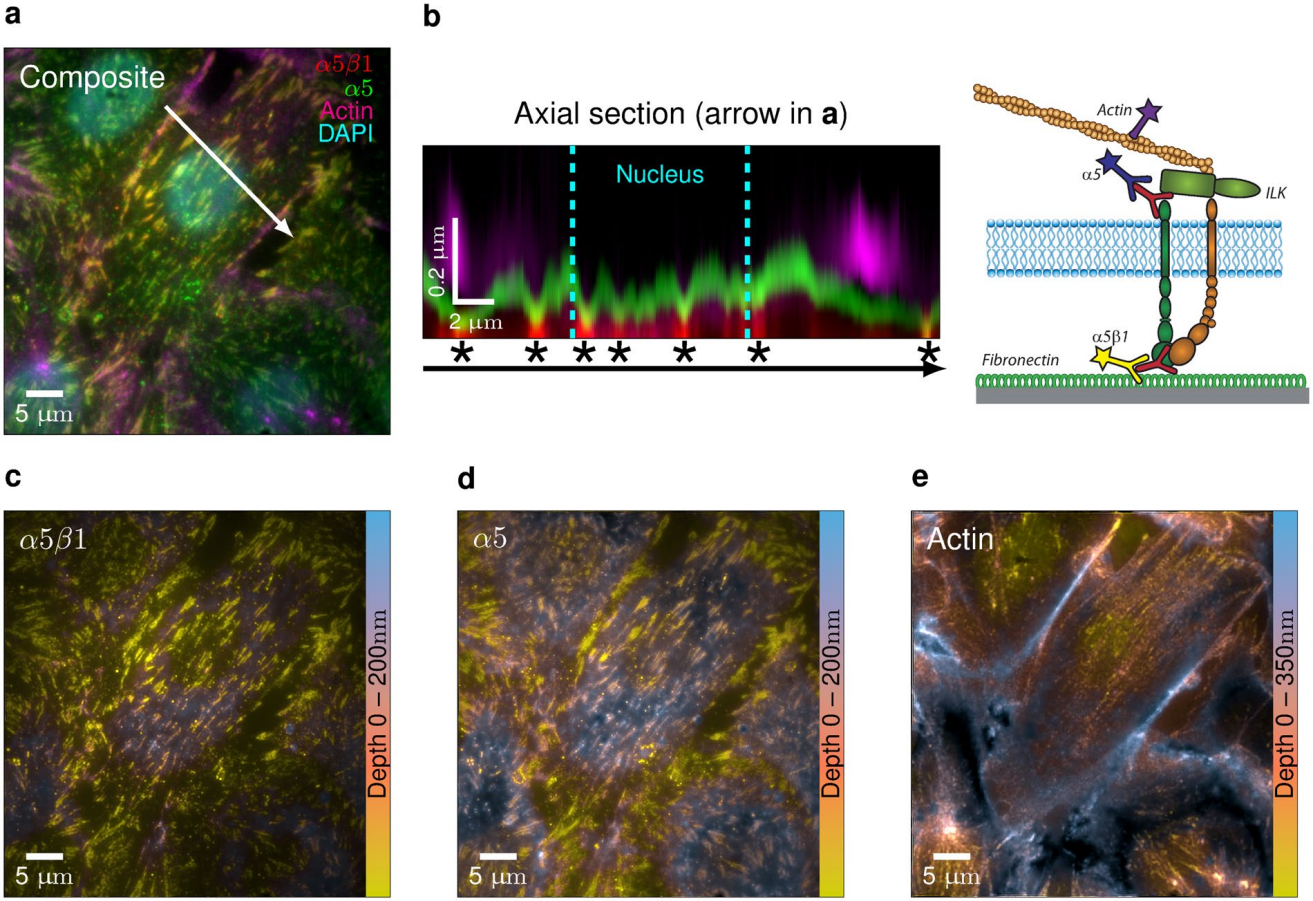
Nanometric axial resolution of integrin *α*5*β*1. Localization of integrin *α*5*β*1 in endothelial cells (4 h after plating on a fibronectin-coated coverslip) was detected using an antibody directed against the intracellular domain of the *α*5 subunit (green) and an antibody against an extracellular epitope of the *α*5*β*1 heterodimer. F-actin was detected with phalloidin (magenta) and nuclei with DAPI (cyan). (**a**) A classical TIRF image of merged channels shows co-localization (yellow) of integrin (in/out) staining at adhesion sites. (**b**) (left) An XZ section from reconstructed stacks along the arrow in (**a**) shows the relative axial location of *α*5*β*1 (out/red), *α*5 (in/green) and F-actin (magenta). Dashed lines indicate the positioning of the nucleus. Asterisks mark adhesion sites. (right) Schematic representation of the *α* and *β* subunits of integrin *α*5*β*1 in an extended-open conformation on a fibronectin-coated substrate and the different secondary antibodies used for stainings. ILK is an adaptor protein involved in linking integrin cytoplasmic tails to the actin cytoskeleton (**c**–**e**) Color-coded height maps of the reconstructions of *α*5*β*1 (**c**), *α*5 (**d**) and F-actin (**e**).

Dual staining of F-actin with Alexa-488 and Alexa-561 phalloidin confirmed the robustness of the method (cross-correlation showed global drift of less than 8 nm) and its ability to allow precise comparison of the axial location of different stainings. To highlight this, we labeled integrin *α*5*β*1, a transmembrane receptor for fibronectin, with different fluorophores on the extracellular and intracellular domains of the protein. Staining was performed in endothelial cells on immobilized fibronectin with antibodies that recognize an epitope in the ectodomain formed by *α* and *β* subunits of the heterodimeric integrin (anti-*α*5*β*1) and antibodies against the cytoplasmic domain of the *α*5 subunit (anti-*α*5). By classical TIRF microscopy it is not possible to discriminate between stainings of intra- and extra-cellular epitopes of *α*5*β*1 localised in focal adhesions (yellow structures shown in the composite of Fig. 4a). In contrast, intracellular anti-*α*5 staining (in green) can be clearly distinguished from extracellular anti-*α*5*β*1 staining (in red) in the axial section depicted in Fig. 4b (left), corresponding to the reconstructed MA-TIRF stack (400 nm depth stack with 20 nm axial steps), along the arrow in Fig. 4a. In MA-TIRF microscopy, as opposed to confocal microscopy, axial localization represents an absolute value with respect to the coverslip. Thus, the axial height of *α*5 staining was found to be 50 nm to 100 nm greater than that of *α*5*β*1. This result is in agreement with estimated dimensions reported for intact ligand-bound integrins in an extended-open conformation^32^, [and references therein] as schematized in Fig. 4b (right). The integrin is in close proximity to the substrate-bearing coverslip when clustered in focal adhesions (asterisks, Fig. 4b) and beneath actin fibers that flank the nucleus. Vertical localization of external and internal epitopes of the integrin across the ventral surface of cells can be visualized in the depth maps shown in Fig. 4c,d, with colors indicating the Z coordinates (0–200 nm) relative to the coverslip surface. Axial positions of actin-rich structures are highest at the periphery of cells and lowest at the tips of stress fibers linked to integrin *α*5*β*1 in adhesion plaques (Fig. 4e, 0–350 nm depth).

**Figure 5:**
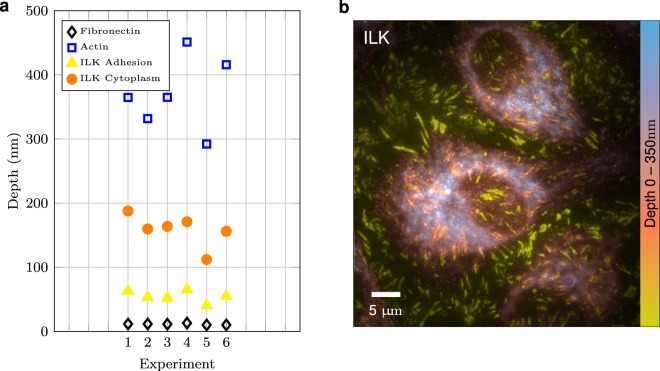
Quantification of ILK altitude. (**a**) Mean altitude of fibronectin, ILK and F-actin in endothelial cells 4 h after plating on immobilized fibronectin. Each abscissa position corresponds to a representative experiment. (**b**) Color-coded depth representation of ILK reconstructions from a representative experiment.

We next determined the axial position of another component of cell-matrix adhesions, ILK, involved in linkage of the *β*1 integrin cytoplamic tail to the actin cytoskeleton, in cells plated on immobilized fibronectin (Fig. 5). It is noteworthy that the adsorbed integrin ligand forms a thin coat with mean altitude less than 20 nm (95%+/−1% of the signal is in the layer 0–20 nm, Fig. 5a). Z-coordinates ILK ranged from 50 nm to 200 nm and displayed a split distribution, above or below 100 nm. It is clear from the color-coded altitude maps that the lower set of molecules corresponds to the presence of ILK in the rim of adhesive structures (called the focal adhesion belt^33^) closest to the fibronectin-coated coverslip (yellow and orange staining, Fig. 5b) and more centrally located adhesions (orange staining, Fig. 5b). The upper population of ILK molecules between 100–200 nm above the coverslip corresponds to diffuse or vesicular staining (pink and blue, Fig. 5b) and thus displayed a greater variation in Z median values. The broadest signal in the Z axis with the highest vertical distribution was obtained for F-actin, with an average height greater than 300 nm across the cell (Fig. 5a).

### Cellular fibronectin assembly beneath endothelial cells

**Figure 6:**
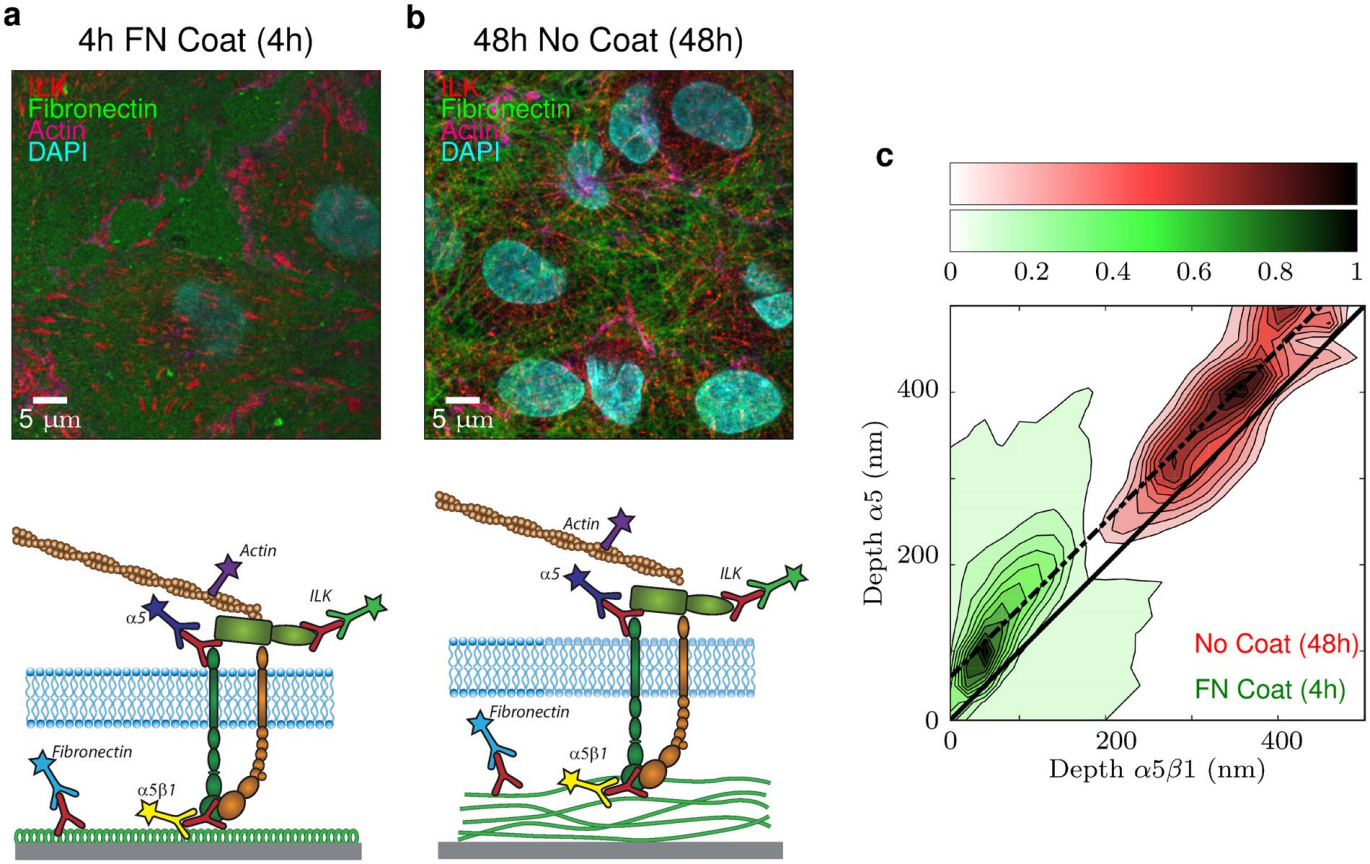
Matrix synthesis. Spinning-disk imaging of endothelial cells either (**a**) 4 h hours after plating on fibronectin (FN) or (**b**) 48 h hours with no coat. F-actin was detected with phalloidin (magenta) and nuclei with DAPI (cyan). Schemes illustrate differences in the topology and thickness of fibronectin that is adsorbed to the coverslip (**a**) or assembled in fibrils following secretion by cells (**b**). (**c**) Correlation of altitude of *α*5 versus *α*5*β*1. The green contours are for cells 4 h after plating and red ones after 48 h. We measured a constant shift of 70 nm (the dashed line in **c**) between *α*5 and *α*5*β*1.

The majority of studies adressing the structure and dynamic regulation of integrins and integrin-based cell adhesions have been performed using cells plated on coverslips coated with immobilized ligands (e.g. plasma fibronectin or fibronectin fragments containing the cell-binding RGD sequence). The fact that cells produce their own matrix proteins and assemble a pericellular matrix, that is fibrillar in the case of fibronectin, is generally overlooked. However, this process is of particular importance in endothelial cells in which basally-directed secretion and assembly of cell-produced fibronectin is required for vascular remodeling during development and in diseased states^34–36^. As illustrated in the spinning disk images of Fig. 6a,b, the topologies of adsorbed plasma fibronectin (Fig. 6a) and fibrillar cell-derived fibronectin (Fig. 6b) beneath endothelial cells are drastically different. Distances between the coverslip and the ventral surface of cells spread on these substrates vary as well. Using our MA-TIRF system, not only can the organization of fibronectin beneath cells be visualized, but the thickness of the matrix deposited by cells and the Z-position of associated cellular proteins can be measured. We previously showed that ILK is required for the maturation of *α*5*β*1-based focal adhesions into fibrillar matrix-forming adhesions associated with the assembly of secreted fibronectin^25,34^. Thus 4 h after plating cells on adsorbed fibronectin, ILK was mostly localized in focal adhesions less than 100 nm from the coverslip (Figs 5 and 6a). However, after 48 h in cells on endogenously-produced fibronectin, both ILK and *α*5*β*1 integrin localized in fibrillar adhesions. These *α*5*β*1-based matrix-assembly structures were higher than 350 nm above the coverslip, as determined by *α*5*β*1 staining (Fig. 6c). The observed difference in depth of the integrin between 4 h and 48 h, schematized in Fig. 6b (bottom), is related to the thickness of the fibronectin matrix. The correlative histogram of Fig. 6c reveals a parallel shift of approximately 70 nm between intra- and extra-cellular *α*5*β*1 epitopes (corresponding to their consistent difference in altitude at 4 h and 48 h). This difference is of the same order of magnitude as the vertical separation observed between intra- and extra-cellular stainings of integrin *α*5*β*1 in cells plated on immobilized fibronectin. Altogether these results highlight the importance of providing extended depth measurements for the study of adhesion events at the ventral surface of cells and the advantage of our MA-TIRF, with respect to classical TIRF microscopy.

## Discussion

Multi-angle TIRF microscopy is a method of choice for the observation of subcellular biological processes. Our custom MA-TIRF setup, together with the proposed calibration pipeline and the dedicated reconstruction algorithm, allows for fast multi-channel tri-dimensional imaging of the basal surface of cells with nanoscale axial resolution. Equipped with fast galvanometric mirrors for controlling the incident angle, the proposed system benefits from a temporal resolution faster than 1s per channel, which makes it compatible with live imaging. Moreover, the reconstruction of a (512 × 512 × 15) volume using the proposed algorithm can be performed within 30 s. This running time can be further decreased to 3 s when GPU computation is activated, which opens the door to online reconstruction for live imaging. It is noteworthy that previously proposed iterative approaches for MA-TIRF reconstruction^21,22^ have relied on optimization methods which include nested iterative routines (*i.e*., inner loop). In contrast, the proposed splitting strategy of the optimization problem allows to deploy an ADMM algorithm for which each step is solved exactly and in a direct way (no inner loop). This allows the reduction of the computational burden per iteration. For instance, the reconstruction (50 iterations) of a (512 × 512 × 20) volume with TV regularization and without considering the convolution operator (for comparison purpose) requires 54 s with the proposed method and 102 s with the algorithm proposed by Zheng *et al*.^22^, while providing the same reconstruction quality. Moreover, a direct adaptation of the previous methods^21,22^ to the joint deconvolution/reconstruction problem would require additional inner loops, leading to a significant increase of the computational cost. Finally, our numerical reconstruction solution is also modular as users can easily (i) provide their own modified TIRF model (ii) switch from one regularizer to another (iii) switch on/off the joint deconvolution and reconstruction. The latter feature of the proposed algorithm, which is completely new, offers the possibility to improve lateral resolution while recovering nanometric axial information. Further, as it is distributed as an open-source software, we also expect it to be a helpful tool for processing data from other MA-TIRF systems.

We demonstrated the potential of the proposed MA-TIRF framework (system + calibration + reconstruction) for the study of fibronectin assembly beneath endothelial cells. Co-localization experiments in which endothelial cells were co-stained with Alexa-561- and Alexa-488-coupled phalloidin, confirmed the ability of the approach to provide coherent axial information from multiple independent channels. This was corrobored by results from others experiments revealing the relative depth of the main actors of cell adhesion and fibronectin matrix assembly with nanometric precision. Importantly, the reconstruction of intra- and extra-cellular stainings of the *α*5*β*1 integrin showed an axial separation in the order of magnitude of the previously estimated length of integrin in an extended-open conformational state. Furthermore this shift was consistently observed in different experiments. Altogether, these results strengthen the significance of MA-TIRF microscopy, and the proposed framework, for shedding light on the process of fibronectin fibrillogenesis as well as other dynamic phenomena near the cell membrane.

## Methods

### Microscopy setup

The microscope has been developed based on a Ti-E inverted microscope base (Nikon) equipped with a 100x/1.49O objective with a fully customized reflexion path. The laser bench (Fig. 2a green part) has been equipped with 405 nm diode laser (Oxxius, Lannion, France), 491 nm DPSS laser (Cobolt, Solna, Sweden), 561 nm DPSS laser (Oxxius) and 640 nm diode laser (Vortran Laser Technology Inc., Sacramento, CA USA). The AOTF (AA optoelectronic, Orsay, France) controls the laser power for DPSS while the two diodes are directly driven. Laser lines are combined taking into account their respective polarization. The laser bench is coupled to the illumination device with a polarization-maintaining optical fiber to optimize the homogeneity of illumination (note that direct coupling is enable for high power purpose).

The illumination device is built around 2 galvanometers (Harvard Apparatus, Les Ulis, France) conjugated with the sample. A first beam expander (4x) let to fill the mirrors while the second one (3x) let to fill the tube lens (200 mm NA/0.15). Fluorescence signal is selected by a quad band dichroic filter for excitation and a filter wheel (Sutter Instruments, Novato CA) for emission. Acquisition is achieved on an EMCCD (Ultra from Andor, Belfast, Northern Ireland). For BFP imaging, a second EMCCD (Ixon2 from Andor) is used.

The system is developed under LabVIEW. Lasers, piezo and galvanometers are driven by DAQ (NI-6731 and NI-6733, National Instruments, Nanterre, France) which store the full experiment design for an acquisition. Rotation rate of the galvanometers (for conical illumination) is adapted to impose a tunable number of rotations per image based on the exposure. Delay time between images imposed by the DAQ is reduced to 1.5 ms based on the devices specificities. This lag is then exploited to transfer the data from the camera and move the galva to their subsequent position. For reconstruction, the acquisition of 10 angles in 1 s or less is usually required. The filter wheel is the slowest element with 40 ms between adjacent filters.

### Cells and culture conditions

Primary cultured bovine aortic endothelial (BAE) cells have been described previously^25,34^. Cells were maintained in DMEM (Invitrogen, Cergy Pontoise, France) supplemented with 5% foetal calf serum (FCS) in a humidified incubator at 37 C with 5% CO2.

### Antibodies and immunofluorescence staining

The following antibodies were used: polyclonal anti-fibronectin, anti-*α*5*β*1 integrin mouse monoclonal (MAB1999) and polyclonal anti-*α*5 integrin subunit (AB1928) from Millipore (Billerica, MA); mouse monoclonal anti-fibronectin (clone 10) and rat monoclonal anti *β*1 integrin (BD Pharmingen™, clone 9EG7) from BD Biosciences (Le Pont de Claix, France); and mouse monoclonal anti-ILK (clone 65.1.9) antibody from Merck Millipore (Molsheim, France). Fluorescently-labelled (Alexa Fluor 488 and 564-conjugated) secondary antibodies, Alexa Fluor 647-conjugated phalloidin, and DAPI (4′,6-diamidino-2-phénylindole) were purchased from Invitrogen (Eugene, Oregon, USA). For immunofluorescence staining, cells were seeded on glass coverslips #1.5. Where indicated, the coverslips were coated with 10 μg/ml plasma fibronectin in phosphate-buffered saline (PBS) for 45 min at 37 °C. At the indicated time after plating, cells were fixed in 3% paraformaldehyde/3% sucrose and permeabilised with 0.2% Triton X-100. After staining, the coverslips were mounted directly in PBS.

### Reconstruction algorithm

From a set of TIRF measurements ${\bf{g}}\in {{\mathbb{R}}}^{{N}_{{\rm{xy}}}\times M}$ acquired with incident angles ${({\alpha }_{m})}_{m\in \mathrm{[1}\ldots M]}$, we aim at estimating the 3D volume *f* in (1). To that end, we first discretize *f* as ${\bf{f}}\in {{\mathbb{R}}}^{{N}_{{\rm{xy}}}\times {N}_{{\rm{z}}}}$ according to a chosen number *N*_*z*_ of *z*-positions. Then, we define the discrete version of model (1) as

3 $${{\bf{g}}}_{m}={\bf{T}}{\bf{H}}{\bf{f}}+{\bf{b}}\,\forall m\in \mathrm{[1}\ldots M],$$

where ${\bf{H}}:{{\mathbb{R}}}^{{N}_{{\rm{xy}}}\times {N}_{{\rm{z}}}}\to {{\mathbb{R}}}^{{N}_{{\rm{xy}}}\times {N}_{{\rm{z}}}}$ convolves each z-slice of the volume **f** with a two-dimensional PSF, and ${\bf{T}}:{{\mathbb{R}}}^{{N}_{{\rm{x}}{\rm{y}}}\times {N}_{{\rm{z}}}}\to {{\mathbb{R}}}^{{N}_{{\rm{x}}{\rm{y}}}\times M}$ defines the discrete TIRF operator that computes TIRF acquisitions of **f** for the incidents angles ${({\alpha }_{m})}_{m\in \mathrm{[1}\ldots M]}$. The decoupling of the operators **T** and **H** in (3) comes from the fact that we consider a two-dimensional PSF $\tilde{h}=h(\cdot ,{z}_{{\rm{fp}}})$. This is a reasonable approximation because, due to the fast decay of the evanescent field, only a thin layer of the sample is excited on which the PSF is close to be constant along the axial direction (see Supplementary Fig. 2). Finally, the background signal **b** is considered spatially constant and is estimated from a region of the acquisitions which does not contain any biological structures.

We then consider a variational approach of the inverse problem through the optimization problem

4 $$\hat{{\bf{f}}}={\rm{\arg }}\mathop{{\rm{\min }}}\limits_{{\bf{f}}\in {{\mathbb{R}}}^{{N}_{{\rm{xy}}}\times {N}_{{\rm{z}}}}}\,(\frac{1}{2}{\Vert {\bf{T}}{\bf{H}}{\bf{f}}-{\bf{g}}\Vert }_{2}^{2}+\mu R({\bf{L}}{\bf{f}})+{i}_{\geqslant 0}({\bf{f}})),$$

where $R({\bf{L}}\cdot )$ can be the Hessian Shatten-norm regularizer^29^ (combination of the mixed norm Shatten (order 1) − ${\ell }_{1}$ and the Hessian operator **L**), or the total-variation regularizer^30^ (combination of the mixed norm ${\ell }_{\mathrm{2,1}}$ and the gradient operator ${\bf{L}}=\nabla$). Finally, $\mu  > 0$ is an hyper-parameter balancing between data-fidelity and regularization and ${i}_{\geqslant 0}({\bf{f}})=\mathrm{\{0}\,{\rm{if}}\,{\bf{f}}\in {{\mathbb{R}}}_{\geqslant 0}^{{N}_{{\rm{xy}}}\times {N}_{{\rm{z}}}},\,+\infty \,{\rm{otherwise}}\}$ enforces the solution to be nonnegative.

We solve problem (4) using the popular alternating direction method of multipliers (ADMM)^28^, or more precisely the simultaneous-direction method of multipliers (SDMM)^37,38^ because the objective (4) is the sum of more than two functions. This algorithm allows to decompose the initial problem into a series of subproblems which can be efficiently solved. By introducing the auxiliary variables ${({{\bf{u}}}_{i})}_{i\in \mathrm{[1}\ldots \mathrm{3]}}$, we split the problem as follows

5 $$\begin{array}{rcl}(\hat{{\bf{f}}},\,{\hat{{\bf{u}}}}_{1},\,{\hat{{\bf{u}}}}_{2},\,{\hat{{\bf{u}}}}_{3}) & = & {\rm{\arg }}\mathop{{\rm{\min }}}\limits_{{\bf{f}},{{\bf{u}}}_{1},{{\bf{u}}}_{2},{{\bf{u}}}_{3}}\,(\frac{1}{2}{\Vert {\bf{T}}{{\bf{u}}}_{1}-{\bf{g}}\Vert }_{2}^{2}+\mu R({{\bf{u}}}_{2})+{i}_{\geqslant 0}({{\bf{u}}}_{3})),\\ {\rm{s}}{\rm{.t}}{\rm{.}}\,{{\bf{u}}}_{1} & = & {\bf{H}}{\bf{f}},\,{{\bf{u}}}_{2}={\bf{L}}{\bf{f}},\,{{\bf{u}}}_{3}={\bf{f}}\mathrm{.}\end{array}$$

The ADMM iterations to solve (5) are summarized in the *Supplementary Algorithm 1*. The decoupling of the spatial convolution and the TIRF excitation in (3) allows us to split according to **u**_1_ = **Hf** and to deploy an efficient (direct, no need for inner iterations) computation for each sub-problems (see *Supplementary Information*).

### Algorithm implementation and hardware ressources

The proposed reconstruction method has been inplemented with MATLAB (The MathWorks Inc., Natick, MA, 2000) within the framework of the GlobalBioIm library^39^. Reconstructions have been performed on a Dell Alienware computer (Intel Core i7-7820X processor). GPU reconstructions were made using a Nvidia (GEFORCE GTX 1080 Ti) graphic card.

### Image visualization

Three-dimensional reconstructed volumes are visualized using a color-coded depth representation. The image intensity in each z-slice of the volume is multiplied by the corresponding element of a isolum color map^40^, as shown in the Supplementary Fig. 1. The resulting volume is then averaged along the axial direction to produce a color-coded depth map of the sample. This representation is particularly well suited for the visualization of such thin volumes.

### 2D histograms

The 2D histograms in Figs 3d and 6c represent the relative depth between two proteins. Let ${R}^{1}\in {{\mathbb{R}}}^{{N}_{{\rm{xy}}}\times {N}_{{\rm{z}}}}$ and ${R}^{2}\in {{\mathbb{R}}}^{{N}_{{\rm{xy}}}\times {N}_{{\rm{z}}}}$ be the reconstructed volumes of two channels, respectively. Then, we define the mean depth maps ${D}^{1}\in {{\mathbb{R}}}^{{N}_{{\rm{xy}}}}$ and ${D}^{2}\in {{\mathbb{R}}}^{{N}_{{\rm{xy}}}}$ such that $\forall i\in \mathrm{[1}\ldots {N}_{{\rm{xy}}}]\,{D}_{i}={\rm{round}}\,({\sum }_{j\mathrm{=1}}^{{N}_{{\rm{z}}}}\,j\times {R}_{ij}/{\sum }_{j\mathrm{=1}}^{{N}_{{\rm{z}}}}\,{R}_{ij})\times {\delta }_{z}$, where ${\delta }_{z}$ denotes the axial discretization step (nm) of the reconstructed volume *R*. The 2D histogram *H* is then obtained by running through the mean depth maps *D*^1^ and *D*^2^ (*i.e*., looping over $i\in \mathrm{[1}\ldots {N}_{{\rm{xy}}}]$) and setting $H({D}_{i}^{1},\,{D}_{i}^{2})=H({D}_{i}^{1},\,{D}_{i}^{2})+\sqrt{({\sum }_{j\mathrm{=1}}^{{N}_{{\rm{z}}}}\,{R}_{ij}^{1})\times ({\sum }_{j\mathrm{=1}}^{{N}_{{\rm{z}}}}\,{R}_{ij}^{2})}$. Finally, the 2D histogram *H* is normalized so as its maximal value is 1.

### Accession codes

The open source software is provided at https://github.com/esoubies/MA-TIRF_Reconstruction.

## Electronic supplementary material

Supplementary Information

## Data Availability

The datasets generated and/or analysed during the current study are available from the corresponding author on reasonable request.
