# Supplementary material for: Nanometric axial resolution of fibronectin assembly units achieved with an efficient reconstruction approach for multi-angle-TIRF microscopy
Source: Sci Rep. 2019 Feb 13;9:1926. doi: 10.1038/s41598-018-36119-3 (PMC6374485; doi:10.1038/s41598-018-36119-3)
Supplement: Supplementary file 1 — Supplementary Information [file 41598_2018_36119_MOESM1_ESM.pdf]

Supplementary Information: Nanometric axial resolution of fibronectin assembly units achieved with an efficient reconstruction approach for multi-angle-TIRF microscopy

Emmanuel Soubies, Agata Radwanska, Dominique Grall, Laure Blanc-Féraud, Ellen Van Obberghen-Schilling and Sébastien Schaub

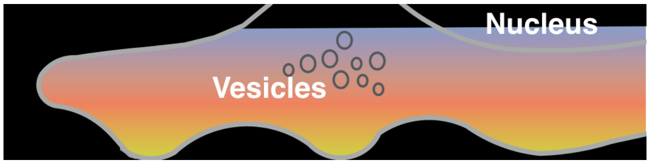

Supplementary Figure 1. Color-coded depth representation for visualization. In this paper, visualization of three-dimensional reconstructed volumes are made by applying a isolum color map[1] in the axial direction as exemplified by this scheme.

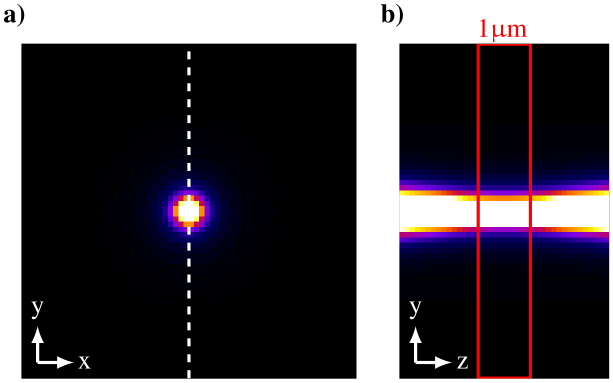

Supplementary Figure 2. Born and Wolf PSF generated using the PSF-generator[2]. Lateral (a) and axial (b) sections. The red rectangle represents an layer of 1 µm depth around the focal plane where the PSF can be considered constant along z.

TIRF theory

In TIRF microscopy, fluorescent probes are excited by an evanescent wave produced in the total internal reflection regime. The fast decay of this wave at the vicinity of the glass coverslip gives access to an unique sectioning capability in comparison with other fluorescent microscopy techniques. Given a tilted illumination with angle α, the decay of the excitation field is governed by an exponential law in the axial direction[3–5]

$$I(z,\alpha) = I_0(\alpha)\exp\left(-zp(\alpha)\right), \tag{1}$$

where

$$p(\alpha) = \frac{4\pi n_i}{\lambda_{exc}}\left(\sin^2(\alpha) - \sin^2(\alpha_c)\right). \tag{2}$$

characterizes the inverse of the penetration depth of the evanescent wave. Here, λ_{exc} is the wavelength of the excitation light, n_i is the refractive index of the incident medium (*e.g.,* glass/oil), and $\alpha_c = \text{asin}(n_t/n_i)$ defines the critical angle with n_t the refractive index of the transmitted medium. The intensity at the interface I_0 (*i.e.,* for $z = 0$) depends on the polarization of the

incident beam. From Fresnel's equations, one can derive its expression for p-polarized and s-polarized light

$$I_0^s(\alpha) = \frac{4\cos^2(\alpha)}{1-n^2}, \tag{3}$$

$$I_0^p(\alpha) = \frac{4\cos^2(\alpha)(2\sin^2(\alpha)-n^2)}{n^4\cos^2(\alpha)+\sin^2(\alpha)-n^2}, \tag{4}$$

where $n = n_t/n_i$. For further details, we refer the reader to the very instructive paper by Martin-Fernandez *et al*[5]. Finally, the total intensity I_0 is well described by a linear combination of I_0^p and I_0^s. Because the microscope uses an azimuthal rotation of the laser beam in order to homogenize the the excitation field and reduce the distortions due to diffusion and diffraction[6,7], it can be shown that[8] $I_0 \simeq \frac{3}{4}I_0^s + \frac{1}{4}I_0^p$.

Given an incident angle $\alpha \in (\alpha_c, \alpha_{\max}]$, where $\alpha_{\max} = \operatorname{asin}(\mathrm{NA}/n_i)$ is the maximal angle that can be used with an objective-based TIRF system, the measured image $\mathbf{g}(\alpha) \in \mathbb{R}^{N_{\mathrm{xy}}}$ is linked to the fluorophore density $f : \Omega \times \mathbb{R}_{\geqslant 0} \to \mathbb{R}_{\geqslant 0}$ through the model

$$[\mathbf{g}(\alpha)]_i = I_0(\alpha) \int\limits_{\mathbf{r}\in\Omega_i} \int\limits_{\mathbf{r}'\in\Omega} \int\limits_{z=0}^{+\infty} h(\mathbf{r}-\mathbf{r}', z - z_{\mathrm{fp}}) \exp(-zp(\alpha)) f(\mathbf{r}', z)\, \mathrm{d}z\, \mathrm{d}\mathbf{r}'\, \mathrm{d}\mathbf{r} + b_i,\ \forall i \in [1 \dots N_{\mathrm{xy}}]. \tag{5}$$

Here N_{xy} denotes the number of pixels of the acquired image and $\Omega_i \subset \Omega$ is the region of the image domain $\Omega \subset \mathbb{R}^2$ that corresponds to the ith pixel, the vector $\mathbf{r} = (x, y) \in \Omega$ represents lateral variables and $h : \Omega \times \mathbb{R} \to \mathbb{R}_{\geqslant 0}$ stands for the three-dimensional point spread function (PSF) of the system. Finally, z_{fp} is the axial coordinate of the focal plane and $\mathbf{b} \in \mathbb{R}^{N_{\mathrm{xy}}}$ models the background signal which is independent of the incident angle α. Because of the fast decay of the evanescent field, only a thin layer of the sample is excited on which the PSF is close to be constant along the axial direction (see Supplementary Figure 2). Hence, we consider a two-dimensional PSF $\tilde{h} = h(\cdot, z_{\mathrm{fp}})$ which allows to decouple the spatial convolution and the TIRF excitation

$$[\mathbf{g}(\alpha)]_i = I_0(\alpha) \int\limits_{z=0}^{+\infty} \exp(-zp(\alpha)) \int\limits_{\mathbf{r}\in\Omega_i} \int\limits_{\mathbf{r}'\in\Omega} \tilde{h}(\mathbf{r}-\mathbf{r}') f(\mathbf{r}', z)\, \mathrm{d}\mathbf{r}'\, \mathrm{d}\mathbf{r}\, \mathrm{d}z + b_i,\ \forall i \in [1 \dots N_{\mathrm{xy}}]. \tag{6}$$

Proposed algorithm and closed-form expression of each subproblem

Algorithm 1 SDMM for solving the inverse problem.

Require: $\mathbf{f}^0 \in \mathbb{R}^{N_{\mathrm{xy}} \times N_{\mathrm{z}}}$, $\rho_D > 0$, $\rho_R > 0$, $\rho_P > 0$

$\mathbf{u}_1^0 = \mathbf{H}\mathbf{f}^0$

$\mathbf{u}_2^0 = \mathbf{L}\mathbf{f}^0$, $\mathbf{u}_3^0 = \mathbf{f}^0$

$\mathbf{w}_i^0 = \mathbf{u}_i^0$, $\forall i \in [1 \dots 3]$

$\mathbf{C} = \rho_D \mathbf{H}^T\mathbf{H} + \rho_R \mathbf{L}^{\mathbf{T}}\mathbf{L} + \rho_P \mathbf{I}$

$k = 0$

while (not converged) **do**

$\quad \mathbf{u}_1^{k+1} = \operatorname{prox}_{\frac{1}{2\rho_D}\|\mathbf{T}\cdot - \mathbf{g}\|_2^2}\left(\mathbf{H}\mathbf{f}^k + \mathbf{w}_1^k/\rho_D\right)$

$\quad \mathbf{u}_2^{k+1} = \operatorname{prox}_{\frac{1}{\rho_R}R}\left(\mathbf{L}\mathbf{f}^k + \mathbf{w}_2^k/\rho_R\right)$

$\quad \mathbf{u}_3^{k+1} = \operatorname{prox}_{i_{\geqslant 0}}\left(\mathbf{f}^k + \mathbf{w}_3^k/\rho_P\right)$

$\quad \mathbf{f}^{k+1} = \mathbf{C}^{-1}\left(\rho_D \mathbf{H}^T\left(\mathbf{u}_1^{k+1} - \mathbf{w}_1^k/\rho_D\right) + \rho_R \mathbf{L}^T\left(\mathbf{u}_2^{k+1} - \mathbf{w}_2^k/\rho_R\right) + \rho_P\left(\mathbf{u}_3^{k+1} - \mathbf{w}_3^k/\rho_P\right)\right)$

$\quad \mathbf{w}_1^{k+1} = \mathbf{w}_1^k + \rho_D(\mathbf{H}\mathbf{f}^{k+1} - \mathbf{u}_1^{k+1})$

$\quad \mathbf{w}_2^{k+1} = \mathbf{w}_2^k + \rho_R(\mathbf{L}\mathbf{f}^{k+1} - \mathbf{u}_2^{k+1})$

$\quad \mathbf{w}_3^{k+1} = \mathbf{w}_3^k + \rho_P(\mathbf{f}^{k+1} - \mathbf{u}_3^{k+1})$

$\quad k = k+1$

end while

Proximity operators From line 7 to line 9 in Alg. 1, proximity operators of three functionals have to be evaluated. All of them admit closed form expressions which can be computed efficiently. Given $g : \mathbb{R}^N \to \mathbb{R}$, its proximal mapping is given by[9]

$$\mathrm{prox}_g(\mathbf{z}) = \arg\min_{\mathbf{x}\in\mathbb{R}^N} \left(\frac{1}{2}\|\mathbf{x}-\mathbf{z}\|_2^2 + g(\mathbf{x})\right). \quad (7)$$

For $i_{\geqslant 0}$, one can easily see that it corresponds to a projection onto the set of positive vectors, that is $\mathrm{prox}_{i_{\geqslant 0}}(\mathbf{z}) = \max(\mathbf{0},\mathbf{z})$ with component-wise operations. The proximity operator of $R = \|\cdot\|_{S_1,1}$ can be computed from the sigular-value decomposition of the Hessian matrix (3×3) at each voxel.[10] Considering the popular TV regularizer[11] would involves $R = \|\cdot\|_{2,1}$ which also admits a closed form proximity operator.[12] Finally,

$$\mathrm{prox}_{\frac{\gamma}{2}\|\mathbf{T}\cdot-\mathbf{g}\|_2^2}(\mathbf{z}) = (\gamma\mathbf{T}^T\mathbf{T}+\mathbf{I})^{-1}(\gamma\mathbf{T}^T\mathbf{g}+\mathbf{z}), \quad (8)$$

where $(\gamma\mathbf{T}^T\mathbf{T}+\mathbf{I})$ is a block diagonal matrix with identical blocks $\mathbf{B}\in\mathbb{R}^{N_z\times N_z}$. Hence, we only need to compute $\mathbf{B}^{-1}$ once and the overall cost of the computation of $\mathrm{prox}_{\frac{\gamma}{2}\|\mathbf{T}\cdot-\mathbf{g}\|_2^2}$ then consists in applying $\mathbf{B}^{-1}$ to each $\mathbf{f}_{i\cdot}\in\mathbb{R}^{N_z}$, $\forall i\in[1\ldots N_{\mathrm{xy}}]$. This is made possible due to the specific structure of the TIRF operator $\mathbf{T}$ which acts independently to each lateral position $i\in[1\ldots N_{\mathrm{xy}}]$.

The linear step of Alg.1 Besides computing the aforementioned proximity operators, the linear step at line 10 of Alg. 1 can often constitute a non-trivial and time-consuming operation (if nested iterations are required). Here, it amounts to invert the linear operator

$$\mathbf{C} = \rho_D\mathbf{H}^T\mathbf{H} + \rho_R\mathbf{L}^\mathbf{T}\mathbf{L} + \rho_P\mathbf{I}. \quad (9)$$

Considering either the Hessian or the gradient operator for $\mathbf{L}$ (with circular boundary conditions), $\mathbf{C}$ is a convolution operator and can thus be inverted in a direct way in Fourier domain.

References

1. Geissbuehler, M. & Lasser, T. How to display data by color schemes compatible with red-green color perception deficiencies. *Opt. express* **21**, 9862–9874 (2013).
2. Kirshner, H., Aguet, F., Sage, D. & Unser, M. 3-d psf fitting for fluorescence microscopy: implementation and localization application. *J. microscopy* **249**, 13–25 (2013).
3. Axelrod, D. Cell-substrate contacts illuminated by total internal reflection fluorescence. *The J. cell biology* **89**, 141–145 (1981).
4. Axelrod, D. Total internal reflection fluorescence microscopy. *Methods cell biology* **89**, 169–221 (2008).
5. Martin-Fernandez, M., Tynan, C. & Webb, S. A ”pocket guide” to total internal reflection fluorescence. *J. microscopy* **252**, 16–22 (2013).
6. Mattheyses, A. L., Shaw, K. & Axelrod, D. Effective elimination of laser interference fringing in fluorescence microscopy by spinning azimuthal incidence angle. *Microsc. research technique* **69**, 642–647 (2006).
7. Fiolka, R., Belyaev, Y., Ewers, H. & Stemmer, A. Even illumination in total internal reflection fluorescence microscopy using laser light. *Microsc. research technique* **71**, 45–50 (2008).
8. Boulanger, J. *et al.* Fast high-resolution 3D total internal reflection fluorescence microscopy by incidence angle scanning and azimuthal averaging. *Proc. Natl. Acad. Sci.* **111** (2014).
9. Moreau, J.-J. Fonctions convexes duales et points proximaux dans un espace hilbertien. *CR Acad. Sci. Paris Ser. A Math.* **255**, 2897–2899 (1962).
10. Lefkimmiatis, S. & Unser, M. Poisson image reconstruction with hessian schatten-norm regularization. *IEEE transactions on image processing* **22**, 4314–4327 (2013).
11. Rudin, L. I., Osher, S. & Fatemi, E. Nonlinear total variation based noise removal algorithms. *Phys. D: nonlinear phenomena* **60**, 259–268 (1992).
12. Combettes, P. L. & Pesquet, J.-C. A proximal decomposition method for solving convex variational inverse problems. *Inverse problems* **24**, 065014 (2008).
